# Biomechanical testing of implant free wedge shaped bone block fixation for bone patellar tendon bone anterior cruciate ligament reconstruction in a bovine model

**DOI:** 10.1186/1749-799X-5-66

**Published:** 2010-09-02

**Authors:** Charles A Willis-Owen, Trevor C Hearn, Gregory C Keene, John J Costi

**Affiliations:** 1 Sportsmed SA, 32 Payneham Road, Adelaide, Australia; 2 School of Computer Science, Engineering & Mathematics, Flinders University, Adelaide, Australia

## Abstract

**Background:**

The use of an interference fit wedged bone plug to provide fixation in the tibial tunnel when using bone-patellar tendon-bone autograft for anterior cruciate ligament reconstruction offers many theoretic advantages including the potential to offer a more economical and biological alternative to screw fixation. This technique has not been subjected to biomechanical testing. We hypothesised that a wedged bone plug fixation technique provides equivalent tensile load to failure as titanium interference screw fixation.

**Methods:**

In a controlled laboratory setting, anterior cruciate ligament reconstruction was performed in 36 bovine knees using bone-patella-bone autograft. In 20 knees tibial fixation relied upon a standard cuboid bone block and interference screw. In eight knees a wedge shaped bone block with an 11 mm by 10 mm base without a screw was used. In a further eight knees a similar wedge with a 13 mm by 10 mm base was used. Each specimen used a standard 10 mm tibial tunnel. The reconstructions were tested biomechanically in a physiological environment using an Instron machine to compare ultimate failure loads and modes of failure.

**Results:**

Statistical analysis revealed no significant difference between wedge fixation and screw fixation (p = 0.16), or between individual groups (interference screw *versus *11 mm *versus *13 mm wedge fixation) (P = 0.35).

**Conclusions:**

Tibial tunnel fixation using an impacted wedge shaped bone block in anterior cruciate ligament reconstruction has comparable ultimate tensile strength to titanium interference screw fixation.

## Background

The ideal choice of graft for Anterior Cruciate Ligament (ACL) reconstruction is controversial, however bone-patellar tendon-bone (BPTB) autograft is a well-established and appropriate option[1]. The optimal form of graft fixation for BPTB graft remains unclear, with a variety of devices in current use[2,3].

Metallic implants such as interference screws can provide adequate tibial bone block fixation. Titanium implants have been used to reduce problems associated with subsequent magnetic resonance imaging (MRI) and for reasons of biocompatibility. Titanium implants have a number of drawbacks including interference with MRI, cost and the requirement for removal prior to revision surgery, which may need supplemental bone grafting and a two-stage procedure. Bioabsorbable implants have been designed to address some of these issues and have been shown to have similar fixation strengths and clinical results[4-6]. Never the less screw breakage, biocompatibility, tunnel widening and delayed synovitis have been reported as potential areas of concern[7-10]. Screws made from allograft bone have proven more difficult to handle and more expensive but do show complete bony integration at 24 months[11]. Interference screws of any sort can be associated with graft laceration, bone plug advancement and reduced fixation strength due to divergence.

Fixation without the use of any implant is appealing for a number of reasons: cost may be reduced; there are no issues regarding biocompatibility; no factors to hinder osseo-integration; and no removal of implant required in the event of revision surgery, meaning a revision procedure can typically be a single stage event. Press fit fixation for the femoral side of ACL reconstruction has been investigated previously and has been demonstrated to be adequate[12-15]. Press fit fixation on the tibial side has been used with some success based around the formation of a tibial trough in which to place the tibial bone block[15,16]. Neither method is in widespread use due to limitations of these techniques. Both techniques are more invasive and time consuming than implant based fixation and concerns exist with difficulty tensioning the graft and the adequacy of fixation using the tibial trough method[16,17]. We have developed a new technique for tibial fixation based of impaction of a wedge shaped bone block into a cylindrical tunnel. To our knowledge, wedge impaction for tibial fixation has not previously been reported.

The objective of this study was to compare conventional titanium interference screw fixation with a novel implant-free method of tibial fixation for BPTB ACL reconstruction, relying on the interference fit of a wedge shaped bone block. Two different sizes of wedge were compared against a control group using an *in vitro *bovine knee model.

## Methods

Bovine knees are an established and acceptable model for biomechanical studies regarding BTPB ACL reconstruction, and have been used in many previous studies[18-24]. Bovine knees were obtained from an abattoir and specimens were wrapped in moist saline swabs and frozen immediately. Knees were thawed for 12 hours prior to reconstruction. The central 40% of the patellar tendon, and corresponding bone blocks was harvested in a standard technique to produce a graft that was similar to the human BPTB graft with regard to its composition and size. All knees included 20 cm of soft tissue and bone proximal and distal to the joint line.

Bone mineral density of the bovine proximal tibia was measured using the Lunar Expert 1107 machine (MEC Osteoporosis Bone Densitometry, Minster, OH, USA) to ensure it was adequate for BPTB graft fixation.

A power calculation was used to determine the required sample size to obtain a power of 0.8 and an alpha value of 0.05. Based on finding a 10% difference in fixation strength between screw and wedge fixation, 16 specimens in each group were required.

**Figure 1:**
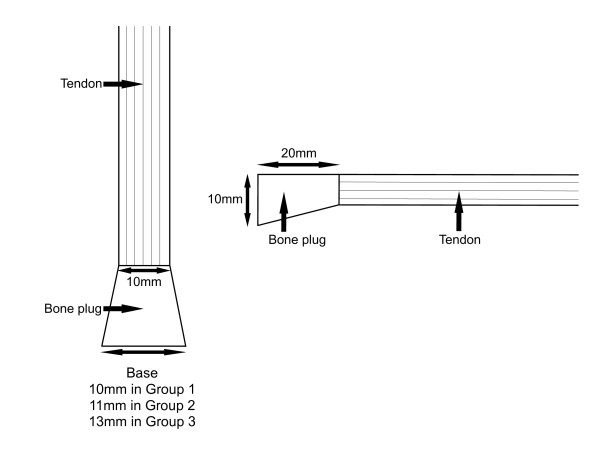
Schematic representation of the wedged portion of the bone-patella tendon-bone autograft

A baseline study of five bovine knees was performed to establish the load to failure of the intact normal ACL in this model. In the control group of 20 knees (group one) a standard rectangular bone block (20 mm long and 10 mm × 10 mm at the free end) was cut from the patella using a power oscillating micro saw. Vernier calipers were used to ensure consistency in the dimensions of all bone blocks to the nearest 0.5 mm. The wedge group of 16 knees was divided into two separate groups with different wedge dimensions. For these groups the bone block was cut in a similar fashion, except for the shape of the patellar bone block. In group two (eight knees) a wedge shape bone block was produced which was 20 mm long and 10 mm × 11 mm at the free end. In group three (eight knees) a broader wedge was fashioned (20 mm long and 10 mm × 13 mm at the free end) (Figure 1). All bone wedges shared the 10 mm wide interface with the tendon to ensure capture of all the tendon fibres.

Following graft harvest the knees were disarticulated by sharp dissection and the proximal tibia mounted in a testing rig. Using a Pro-Trac tibial guide (Smith & Nephew) set at 45° and positioned in a standard fashion over the ACL footprint, a guide wire was passed through the guide and then over-reamed slowly using a 10 mm cannulated reamer (Smith & Nephew). Moist saline swabs were used to remove debris. Soft tissue was dissected from the tibial entrance to prevent snaring.

**Figure 2:**
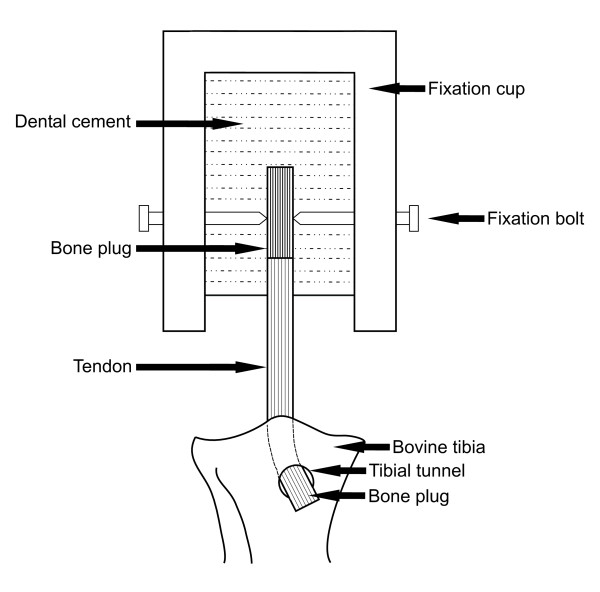
Schematic representation of femoral fixation method.

The graft was inserted in the line of the tunnel under manual tension and the tibial plug impacted as required using a mallet and punch. All grafts were inserted until flush with the anterior tibial cortex. Despite requiring more force for insertion the 13 mm wedge blocks were inserted without significant damage. The femoral plug was secured into the testing apparatus using fixation bolts and dental cement (Vertex, slow self curing cement, Dentimax BV, The Netherlands) (Figure 2).

In group one a standard 9 mm × 20 mm cannulated titanium interference screw (Kurosaka, DePuy) was inserted to provide interference screw fixation in the conventional manner. A guide wire was used to prevent screw divergence. In groups two and three, fixation depended only on the interference fit of the wedge shaped bone plug.

All reconstructed knees were kept moist (in saline packs) and allowed 12 hours standing time to allow for the possible effects of bone stress relaxation before mechanical testing was carried out.

Knees were tested using an Instron model 8511 servo-hydraulic material testing system (Instron Pty. Ltd., High Wycombe, UK). The knee was secured in place using a universal joint, which allowed the ACL to align freely along the line of force. The specimens were maintained in a circulating saline bath environment at 37°C prior to and during testing. The ACL was preconditioned to 220N for 20 cycles at 0.5 Hz using a sinusoidal waveform. Specimens were then loaded to failure at a constant displacement rate of 60 mm/min. The mode of failure and peak loads to failure were recorded.

Wedge groups (group two and group three) were merged and a Student's t-test was performed to compare wedge fixation against screw fixation. In addition a univariate analysis of variance was used to assess the difference between all three groups.

## Results

Bone mineral density in the bovine tibiae ranged from 0.89 to 1.13 grams per cm^2 ^which is comparable to that of patients undergoing ACL reconstruction[25].

The modes of construct failure observed are shown in Table 1. Seven specimens failed at the attachment of the femoral plug to the testing rig. In these specimens data for the maximum load to failure of tibial fixation were not obtained, however it was inferred that the load to failure of tibial fixation was at least as high as that for failure of femoral fixation.

**Table 1:** Count of occurrences for each mode of failure

Group	Tibial	Femoral	Avulsion	Total
Baseline	-	-	5	5

Group 1	13	7*	-	20

Group 2	1	5	2	8

Group 3	2	3	3	8

*These data were not included in further analyses

For the purposes of statistical analysis these data were treated as fixation failures in groups two and three, but censored from the control group. This approach was adopted to avoid artificial reduction of the mean load to failure in the control group, whilst not losing data from the test groups. It potentially therefore underestimated any advantage of groups two and three over the control group, however it ensured that meaningful data was not excluded from analysis. Table 2 summarizes the descriptive statistics and modes of failure for each group.

**Table 2:** Descriptive statistics and mode of failure for each group

Group	Sample size	Mean (N)	Standard Deviation (N)	Range (N)
Baseline	5	501	69.5	387-556

Group 1*	13	409	82.8	270-577

Group 2	8	478	96.3	343-634

Group 3	8	438	124.8	309-678

*femoral failures excluded

With groups two and three merged into a single group for wedge fixation a two-tailed unpaired Student's t-test was performed with no significant difference observed (p = 0.16). To assess difference between all three groups a univariate analysis of variance with a factorial structure of procedure (screw *versus *11-mm graft *versus *13-mm graft) was performed. This analysis revealed that there was no significant effect due to procedure (P = 0.35). Thus the ultimate load to failure of wedge fixation was demonstrated to be at least as equivalent to that achieved with interference screw fixation with a non statistically significant trend for superiority.

## Discussion

This study found that it is possible to achieve an acceptable initial tibial fixation without the need for any implant by using a wedge shaped bone block. The mean load to failure observed for both sizes of wedge shaped bone blocks was equivalent to interference screw control group, and were comparable to the mean load to failure of the native ACL in this model.

The 11 mm wedge of group two did not differ significantly from the 13 mm wedge of group three revealing that an 11 mm wedge is adequate for this technique however the sample sizes for this comparison were small. It is possible that the lack of significant difference here was due to insufficient statistical power. A narrower wedge is preferable since it reduces the amount of bone take from the patella and may be easier to fashion.

The wedge shaped bone block can be cut from the patella in exactly the same manner as a rectangular bone block with by simply diverging the longitudinal patella saw cuts. This procedure requires no further dissection or bone preparation over the use of a rectangular block and interference screw (as opposed to the tibial trough press fit method of tibial fixation). It does not introduce any additional operative time or cost. Less equipment, and fewer operative steps are needed compared to the use of an interference screw.

The cancellous surface of a bone plug, if handled properly remains osteogenic, is easily vascularised, and readily incorporated into host bone. Interference screws are routinely applied to the cancellous surface of the bone plugs to maximise graft fixation, however this reduces the contact area between cancellous surfaces of the bone plug and the tibia[26]. This new method allows a greater cancellous to cancellous contact area and so may be expected to provide early and more robust integration of the bone plug.

The technique does have some potential limitations. Tibial fixation must precede femoral fixation, and tensioning of the graft must take place from the femoral side. In order to overcome these obstacles we advocate the use of transfixing pin fixation for the femoral side after impaction of the wedge bone block into the tibia and appropriate tensioning. Any excess length of graft must be accommodated on the femoral side, and impacting the wedge into the tibial tunnel can compensate for a short graft. If for any reason the tibial fixation is deemed to be inadequate it can be easily augmented with an interference screw in the conventional manner.

Our experimental setup had a number of limitations. Firstly the bovine model used is not a perfect representation of living human tissue and the loads to failure observed in our baseline group were not comparable to those observed in human tissue. Our recorded loads to failure were of a similar value to that of the native ACL recorded in the baseline group suggesting that our comparisons are valid. Bovine knees have shown to be a superior model for ACL reconstruction to that of elderly cadaveric human tissue[19], and the acquisition of young human cadaveric knees is problematic and costly. Secondly, despite our best efforts a number of samples failed at the testing rig - femoral bone block interface, meaning that the tibial fixation was not tested to failure in these cases. For the purposes of statistical analysis failure on the testing rig - femoral bone block interface was treated as failure of the construct for groups two and three thus leading to an underestimate of the true fixation load to failure, and data was censored for the screw fixation group in order avoid under estimating the fixation strength achieved. Thus our analysis tended to under estimate any superiority of the wedged bone block method. Repetition of the study using a more robust fixation system would be informative.

The measurement of ultimate load to failure is one accepted method for evaluating ACL graft fixation and is widely used in the literature[27-29]. It is known that there are changes in ACL orientation with cyclical loading[30]. It would be informative to test this fixation method with cyclical loading tests.

Various methods of implant free tibial fixation have been reported in the past. Bernard et al (1992) developed a technique using a bone plug fixed in the femur and tibia without screws[31]. A modification of this technique was reported by Georgoulis et al (1997) with good mid term results[32]. An anterior trench was used allowing plug insertion then the cortical roof was replaced and secured with trans-osseous pins. An alternative technique was described by Boszotta (2003) involving the use of circular reamers to harvest cylindrical bone plugs[33]. Wedge shaped plugs have been shown to be successful for femoral fixation in both biomechanical studies and clinical trials[14,34,35].

## Conclusions

This novel technique has been shown to produce sound immediate tibial fixation for BPTB grafts. There is the potential for prompt direct bone integration to provide durable fixation. It avoids the pitfalls associated with metallic or bioabsorbable fixation devices, simplifies revision procedures, and requires no additional incisions dissection or instrumentation. In the rare event of difficulties attaining fixation, screw augmentation is a simple additional step. Clinical studies using this method of fixation would be of interest.

## Competing interests

The authors declare that they have no competing interests.

## Authors' contributions

CWO analylsed results, and wrote the manuscript, TH and JC carried out the lab work, GK designed the technique, GK and JC designed the study. All authors read and approved the final manuscript.
